# Clinical Ethics Support for Healthcare Personnel: An Integrative Literature Review

**DOI:** 10.1007/s10730-017-9325-4

**Published:** 2017-06-09

**Authors:** Dara Rasoal, Kirsti Skovdahl, Mervyn Gifford, Annica Kihlgren

**Affiliations:** 1 School of Health and Medical Sciences, Örebro University, Fakultetsgatan 1, SE - 701 82 Örebro, Sweden; 2 Department of Nursing and Health Sciences, University College in Southeast Norway, Drammen, Norway

**Keywords:** Ethics, Health personnel, Moral case deliberation, Ethics consultation, Ethics committees, Ethics rounds, Ethical reflection

## Abstract

This study describes which clinical ethics approaches are available to support healthcare personnel in clinical practice in terms of their construction, functions and goals. Healthcare personnel frequently face ethically difficult situations in the course of their work and these issues cover a wide range of areas from prenatal care to end-of-life care. Although various forms of clinical ethics support have been developed, to our knowledge there is a lack of review studies describing which ethics support approaches are available, how they are constructed and their goals in supporting healthcare personnel in clinical practice. This study engages in an integrative literature review. We searched for peer-reviewed academic articles written in English between 2000 and 2016 using specific Mesh terms and manual keywords in CINAHL, MEDLINE and Psych INFO databases. In total, 54 articles worldwide described clinical ethics support approaches that include clinical ethics consultation, clinical ethics committees, moral case deliberation, ethics rounds, ethics discussion groups, and ethics reflection groups. Clinical ethics consultation and clinical ethics committees have various roles and functions in different countries. They can provide healthcare personnel with advice and recommendations regarding the best course of action. Moral case deliberation, ethics rounds, ethics discussion groups and ethics reflection groups support the idea that group reflection increases insight into ethical issues. Clinical ethics support in the form of a “bottom-up” perspective might give healthcare personnel opportunities to think and reflect more than a “top-down” perspective. A “bottom-up” approach leaves the healthcare personnel with the moral responsibility for their choice of action in clinical practice, while a “top-down” approach risks removing such moral responsibility.

## Introduction

Healthcare personnel frequently face ethically difficult situations in the course of their work and these issues cover a wide range of areas in clinical practice (Åstrom et al. 1995; Beauchamp and Childress 2009; Lindseth et al. 1994; Sørlie et al. 2000; Tabitha et al. 1979) and community home healthcare services (Karlsson et al. 2013). In such situations, healthcare personnel can experience unease or uncertainty (Cohen and Erickson 2006) over what is right or good to do, or there may be disagreement about what should be done. Moreover, some ethical issues can be connected to conflicting interests between healthcare workers and patients and their next-of kin (Beauchamp and Childress 2009; Rasoal et al. 2015); for example, situations where patients do not follow the recommendations of healthcare personnel, such as when patients and healthcare personnel have different opinions regarding what to do (Hermsen and van der Donk 2009; Slettebø and Bunch 2004), or issues that are related to ongoing life-sustaining treatment (Cassel 1984; Schaffer 2007; Silén et al. 2008). At times, healthcare personnel experience distress as a result of ethical issues in patient care (Kälvemark et al. 2004; Pauly et al. 2009).

One way to support healthcare personnel in dealing with these ethical challenges has been through the development of clinical ethics support (CES). CES is defined as the formal or informal provision of advice and support to healthcare personnel on ethical issues arising from clinical practice and patient care within the healthcare setting (Owen 2001; Puntillo et al. 2001; Slowther et al. 2004a). CES is becoming more prevalent with the increased awareness worldwide of the importance of ethical issues in healthcare and with personnel encountering an increasing number of ethical issues in clinical practice (Bartholdson et al. 2015; Doran et al. 2015; Oberle and Hughes 2001; Ulrich et al. 2010).

Philosophical papers and empirical research have led to the development of various approaches to CES that have the goal of supporting healthcare institutions, healthcare personnel,  and patients as well as next-of-kin (Åstrom et al. 1995; Reiter-Theil and Hiddeman 2000). There are no universal norms regarding which approaches should be used to support healthcare personnel in clinical practice. CES approaches can roughly be divided into “top-down” or “bottom-up” perspectives, which can be contrasted in terms of the nature, purpose and goals of the support. Within “top-down” perspectives, an ethical consultant or a group of “experts” has an influential advisory role or act(s) as the primary ethical decision maker, providing advice or recommendations (Aulisio et al. 1998; Crigger 1995; La Puma and Schiedermayer 1991). Those supporting such an approach claim that the ethical issues in healthcare are too complex to be managed by the healthcare personnel themselves. In this vein, personnel facing ethical issues require specialist expertise in the same way that medical doctors need to consult with each other within different specialties (La Puma and Schiedermayer 1991). In contrast, in “bottom-up” approaches to CES, reflection begins with healthcare personnel’s everyday experiences of ethical issues in clinical practice (Hansson 2002). The discussion is facilitated by an ethicist or philosopher, a “facilitator” who has the goal of fostering greater insight among the personnel into ethical considerations rather than focusing on decision-making in any particular case (Hansson 2002; Stolper et al. 2014). Adherents of “bottom up” approaches claim that ethical issues need to be reflected on critically by the healthcare personnel themselves, since they are the only legitimate decision-makers and are morally responsible for the outcomes (Hansson 2002). The facilitator for such an approach is considered to lack the knowledge needed to give advice and make recommendations for the best course of action. The existence of such contrasting approaches leaves the question open regarding which approach can be “the golden middle way” to guide healthcare personnel in clinical practice.

However, to our knowledge, there is a lack of integrative reviews regarding available approaches to ethics support and how different approaches support healthcare personnel deal with ethical issues. It is reasonable to believe that practitioners need some kind of CES reflection that relates to their personal experiences of everyday ethical issues. Therefore, in this paper, we aim to describe which clinical ethics support approaches are available to support healthcare personnel in clinical practice in terms of their construction, functions and goals.

## Method

### Design

This integrative literature review applies a descriptive design using the matrix method  (see Garrard 2010).

### Search Strategy

#### Systematic Search

Electronic databases of CINAHL, MEDLINE and Psych INFO were systematically used to search for relevant peer-reviewed articles. This literature review process was begun by first identifying specific search terms (i.e., indexed search terms) thorough Cinahl headings, Mesh and Thesaurus. A list of possible search terms that could be relevant for the study aim was created. In the database Psych INFO, we used the following suggested search terms: “ethics” AND “health personnel”; in CINAHL, the headings “ethics” OR “ethics committees” AND “health personnel”; in MEDLINE, the Mesh terms “ethics” OR “ethics committees” OR “ethics consultation” OR “clinical ethics” OR “institutional ethics” AND “health personnel” were used.

#### Manual Search

**Fig. 1:**
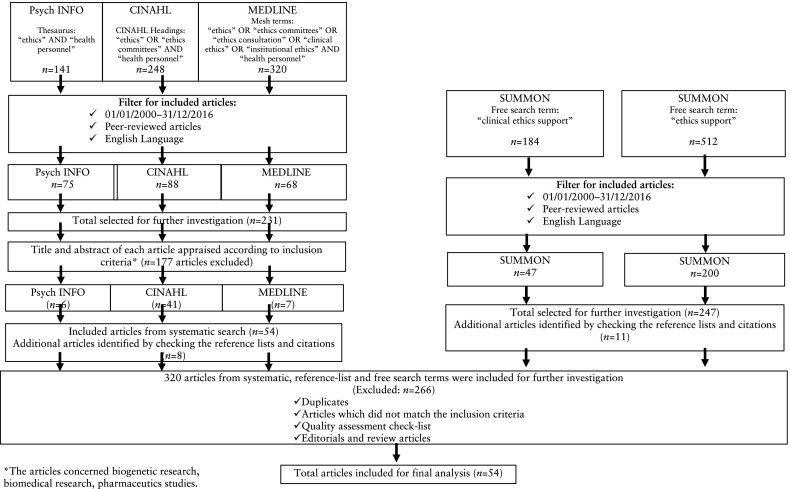
Overview flowchart regarding the search steps and inclusion process

The expertise of two experienced librarians from the university and the university hospital independently assisted in the search for relevant articles together with the first author. The two manual searches conducted in the database Summon used the following search terms: “clinical ethics support” and “ethics support”. A more detailed description of the search strategy is provided in Fig. 1.

### Inclusion and Exclusion Criteria

The selected articles consisted of: (1) empirical studies or theoretical papers, (2) that reflected on ethical issues in health care, (3) and that wrote about established ethics support approaches aimed at supporting healthcare personnel in clinical practice. Articles were excluded if they concerned approaches that only focused on patients and families, research design issues, policy, education, biogenetic research, pharmaceutical studies, or research on animals. Editorials and review articles were also excluded.

### Search Outcome

#### Systematic Search

The systematic search in Psych INFO yielded 141 articles, in CINAHL 248 articles and in MEDLINE 320 articles (Fig. 1). Limiting the search to English-language peer-reviewed journals published in 2000–2016, reduced the total number of articles from the three databases to 231 articles. The titles, and when available the abstracts, were scrutinized by the authors in relation to the inclusion criteria, which resulted in the exclusion of 177 articles and the selection of 54 articles for further investigation. From the systematic search of all three databases, 54 articles were selected for further investigation. After checking the reference lists of the articles and citations, eight additional articles were found.

#### Manual Search

The first manual search using the search term “clinical ethics support” yielded 184 articles. The second manual search using the search term “ethics support” found 512 articles. After applying the inclusion criteria of English-language peer-reviewed articles, published in 2000–2016, the number of articles reduced to 247. Checking the reference lists and citations revealed 11 additional articles.

#### Quality Appraisal

In total, 320 articles from the systematic and manual searches, as well as additional articles that were identified by checking the reference lists and citations, were included for further investigation. Three of the authors read and appraised the articles by reading the titles, and when available the abstracts. We excluded duplicates, articles that did not match the inclusion criteria, editorials and review articles. After the appraisal of the 320 articles, 54 remained for further analysis. Full text was not available for three of the articles, and they had to be purchased. All the articles were discussed among all of the authors in order to reach agreement regarding the content in relation to the research aim. We used a quality assessment check of the included articles (SBU 2014). The included articles were both theoretical papers and empirical research that reflected on ethically difficult situations in health care and how to support health personnel from diverse cultures and countries worldwide.

### Data Analysis

Empirical, qualitative and quantitative studies as well as theoretical papers with various approaches were included. First, the articles were sorted based on the CES approach. Second, a critical review of each article was performed, with particular attention given to the results and conclusions and their relation to the aim provided in the article. Notes were made regarding their content (Table 1). The analysis process was inspired by manifest content analysis (Graneheim and Lundman 2004).Third, the important parts of each article, such as approach, aim, method, results and conclusion, were written up into a matrix (Garrard 2010). Fourth, the results and conclusions of each qualitative, quantitative and theoretical paper were imported into to a new sheet in a word processor so they could be coded. Fifth, the first author performed descriptive coding of the articles’ results and conclusions. Sixth, based on content similarities and differences among the similar approaches, descriptive and manifest categories emerged from the codes. Finally, the results of each category were synthesized into an integrated result. The integration is meant to synthesize and clarify what is known about a target phenomenon (Sandelowski et al. 2006), such as different approaches to CES.

**Table 1:** Literature review matrix over approaches to clinical ethics support

Author/date/title/country/journal	Approach	Methods/sample	Research aim	Results/conclusions
Agich (2001). The question of method in ethics consultation. USA. *The American Journal of Bioethics*	Clinical ethics consultation	Theoretical paper	To describe the rules involved in ethics consultation	The canon of ethics consultation is that set of rules that guides the action, cognition, and perception involved in doing ethics consultation. The discipline of ethics consultation includes the rule-guided actions and behaviors comprising ethics consultation. It also refers to the specific training that produces the type or pattern of action and behavior in question. There is no dearth of proposed models of ethics consultation; but there is little sound methodological ethics consultation in the practical engagement of an ethicist in the care of patients.
Adams (2009). Ethics consultation and ‘facilitated’ consensus. USA. *Journal of Clinical Ethics*	Clinical ethics consultation	Theoretical paper	To use a case to illustrate some potential problems with the standards of the American Society for Bioethics and Humanities as described in the Core Competencies	The Core Competencies is meant to be a blueprint for how ethics consultations are to unfold. But the worry is that the contextual factors to which the Core Competencies defers may not be sufficiently robust to channel moral deliberation to a degree that will forestall complaints that the process of ethics consultation lacks effectiveness and legitimacy.
Aulisio et al. (2000). Health care ethics consultation: Nature, goals, and competencies. USA. *Annals of Internal Medicine*	Clinical ethics consultation	Qualitative, interdisciplinary group discussion over two years of 19 scholars representing diverse fields	To summarize the conclusions of the Task Force Report	The Task Force Report contains nine general conclusions: (1) US social context makes “ethics facilitation” an appropriate approach to ethics consultation; (2) ethics facilitation requires certain core competencies; (3) core competencies can be acquired in various ways; (4) individual consultants, teams, or committees should have the core competencies for ethics consultation; (5) consult services should have policies that address access, patient notification, documentation, and case review; (6) abuse of power and conflicts of interest must be avoided; (7) ethics consultation must have institutional support; (8) evaluation of process, outcomes, and competencies is needed; and (9) certification of individuals and accreditation of programs are rejected.
Aulisio et al. (2009). Clinical ethics consultation and ethics integration in an urban public hospital. USA. *Cambridge* *Quarterly of Healthcare Ethics*	Clinical ethics consultation	Theoretical paper	To describe the evolution of an ethics consultation service at a metro medical center in an urban public hospital, its struggle to thrive, and subsequent revitalization	Ethics consultation utilized a service that increased fourfold over a three-year period, a usage rate maintained since. A key step was its use of an adaptive small-team approach including an ethics consult–care team meeting. These meetings often result in either (1) the dissolution of apparent ethical conflict or uncertainty as lines of communication are opened or (2) clarity on the part of the care team members regarding the next steps they must take in order to address the ethical issues under discussion.
Fox et al. (2007). Ethics consultation in United States hospitals: A national survey. USA. *The American Journal of Bioethics*	Clinical ethics consultation	Quantitative, *n* = 56 phone or questionnaire surveys	To describe the prevalence, practitioners, and processes of ethics consultation in US hospitals	Response rate was 87.4%. Ethics consultation services (ECSs) were found in 81% of all general hospitals in the USA, and in 100% of hospitals with more than 400 beds. Most individuals performing ethics consultation were physicians (34%), nurses (31%), social workers (11%), or chaplains (10%). Only 41% had formal supervised training in ethics consultation. Consultation practices varied widely both within and between ECSs. For example, 65% of ECSs always made recommendations, whereas 6% never did. These findings highlight a need to clarify standards for ethics consultation practices.
Fukuyama et al. (2008). A report on small team clinical ethics consultation programmes in Japan. Japan. *Journal of Medical Ethics*	Clinical ethics consultation	Qualitative, evaluation of educators, researchers from the area of biomedical *n* = 17	To examine the process of evaluating small team clinical ethics consultation services, as well as the strengths and weakness of such programs	In Japan, clinical ethics consultation services should be regarded as supplementary. They concentrate on nationwide educational activities and providing on demand local clinical consultation services with second opinions from an ethical point-of-view. The Clinical Ethics Support and Education Project works as the first and only small team clinical ethics consultation service in Japan.
McClimans et al. (2016). A qualitative study of US clinical ethics services: Objectives and outcomes. USA. *Narrative Inquiry in Bioethics*	Clinical ethics consultation	Qualitative, in-depth interviews with experts*, n* = 19	To explore the views of experts about the objectives and outcomes of a clinical ethics services	*Twelve objectives:* Mediation, counselling, hospitality, empowerment, managing moral distress, improvement of ethical and moral quality of decision and action, education, improvement of critical thinking skills, awareness of ethics, being of service, preventative ethics, and improvement of quality of patient care. *Nine outcomes*: Satisfaction with the processes or expertise of the CES, satisfaction with clinical ethics recommendations, elimination of non–beneficial treatment, productivity, integration, reduction of lawsuits, consensus, transformation of individuals, transformation of institutions. The experts were divided in their emphasis on the kinds of objectives that are most important. In terms of outcomes, experts were concerned with the appropriateness of different proxy and direct measures.
Schochow et al. (2015). Implementation of clinical ethics consultation in German hospitals. Germany. *Science and Engineering Ethics*	Clinical ethics consultation	Qualitative; follow up survey, *n* = 1858 hospitals	Follow-up survey concerning the availability of ethics consultation	The survey revealed that 912 hospitals in all of Germany have at least one form of clinical ethics consultation available. The health care ethics committee is the most frequently implemented structure of clinical ethics consultation.
Tarzian and ASBH Core Competencies Update Task Force (2013). Health care ethics consultation: An update on core competencies and emerging standards from the American Society for Bioethics and Humanities’ Core Competencies Update Task Force. USA. *The American Journal of Bioethics*	Clinical ethics consultation	Theoretical paper	To clarify, revise and expand the content of health care ethics consultation core competencies	Health care ethics consultation is now an integral part of US health care delivery. The assumption that the consultant does not need specific competencies aside from general knowledge and skills has been rejected by the American Society for Bioethics and Humanities. Ethics consultation is a distinctive services that responds to a specific request for assistance, focuses on addressing uncertainty or conflict regarding value-laden concerns and addresses those value-laden concerns through “ethics facilitation”. Those designated to perform the role should have the requisite competencies to address the question or concern appropriately in health care consultation.
Rasmussen (2011). An ethics expertise for clinical ethics consultation. USA. *Journal of Law, Medicine & Ethics*	Clinical ethics consultation	Theoretical paper	To explain the ethical expertise involved in clinical ethics consultation	Ethics expertise concerns a variety of considerations that bear on moral decision making. When a patient, family, or healthcare professional wants guidance on a moral matter, usually they do not want help disciplining themselves to do the right thing. For the most part, they are motivated to do the right thing, but because of the complexity of the situation, the right action is not clear.
Reiter-Theil (2000). Ethics consultation on demand: Concepts, practical experiences and a case study. Germany. *Journal of Medical Ethics*	Clinical ethics consultation	Theoretical paper	To describe experiences from the University Hospital of Freiburg regarding the provision of clinical ethics support	Ethics consultation developed as a consequence of increased ethical awareness, expansion of medical interventions, influence of legal aspects, economic constraints, patients dying in hospital and experiences of ethical conflict related to treatment at the end of life.
Rasmussen (2016). Clinical Ethics consultants are not “ethics” experts-but they do have expertise. USA*. Journal of Medicine and Philosophy*	Clinical ethics consultation	Theoretical paper	To describe clinical ethics consultation and their expertise concerning the right moral answer	Clinical ethics consultation is substantive, which requires a kind of training that other professions undergo, but that is not normatively binding. Opponents of CEC and moral expertise may essentially be objecting to the idea of people who profess to have the right answer in moral situations, because: (1) they hold that there is no such objectively verifiable thing, and (2) this society respects and protects autonomous moral decision-making more highly than correct moral decision-making.
Rwabihama et al. (2010). Ethics committees for biomedical research in some African emerging countries: Which establishment for which independence? A comparison with the USA and Canada. *Journal of Medical Ethics*	Clinical ethics committees	Mixed methods. Questionnaire sent to *n* = 25 countries in Africa and north America and recorded interviews with ethics committees during three months, *n* = 2	To investigate the process of establishing ethics committees and their independence	In total, 22 countries participated in this study, with 20 from Africa and two from North America. The process of establishing ethics committees could affect their functioning and compromise their independence in some African countries and in North America.
Gaudine et al. (2010). Evolution of hospital clinical ethics committees in Canada. Canada. *Clinical Ethics*	Clinical ethics committees	Mixed methods. Questionnaire and open-ended questions. Questionnaires *n* = 265 to all English and French-language Canadian acute care hospitals with 100 or more beds	To investigate the current status of hospital clinical ethics committees and how they have evolved in Canada over the past 20 years	One hundred and five respondents reported that their hospital had a CEC. The majority indicated that the role of the CEC was primarily advisory. 96.2% of respondents reported that attending physicians could refer an issue to the committee. Ethics committees also provided ethics education.
Akabayashi et al. (2008). A five year follow-up national study of ethics committees in medical organizations in Japan. Japan. *HEC Forum*	Clinical ethics committees	Quantitative, participants from the Japanese Association of Medical Sciences 1998 and 2003, *n* = 92 and *n* = 96	To determine the creation and function of ethics committees at medical organizations in Japan, and their general strategies for dealing with ethical problems	The major roles of ethics committees include ethical reviews of research protocols, policy making, and ethical reviews of manuscripts submitted for journal publication.
Aulisio and Arnold (2008). Role of the ethics committee. Helping to address value conflicts or uncertainties. USA. *Medical Ethics*	Clinical ethics committees	Theoretical paper	To address questions about the existence and function of ethics committees	Legal, regulatory and professional forces drove the development of ethics committees. Ethics committees were developed in response to clinical needs for a formal mechanism to address some of the value conflicts and uncertainties that arise in contemporary health care settings.
Borovečki et al. (2010). Developing a model of healthcare ethics support in Croatia. Croatia. *Cambridge Quarterly of Healthcare Ethics*	Clinical ethics committees	Discussion article. Different ethics support related to case studies	To determine what type of ethics support would be suitable for the Croatian health care system	A number of steps need to be taken in order for Croatian ethics committees to develop the kind of robust institutional education programs that can foster and support the ethics case deliberation model: (1) clarification of the selection criteria for committee membership, (2) ethics committees should assume the responsibility of educating healthcare teams as their first priority and, (3) ethics committees should facilitate the creation of a database of cases presenting ethical dilemmas.
Caminiti et al. (2011). Current functions of Italian ethics committees: A cross-sectional study. Italy. *Bioethics*	Clinical ethics committees	Mixed methods. Questionnaire to ethics committees, *n* = 191. Telephone interviews, *n* = 4	To give an overview of the different types of activities of Italian ethics committees and support for ethical discussion at a European level	This study surveys the types of activities carried out by ethics committees: to promote the training, education and information of healthcare staff, patients and families or the public; to advise on the care of individual patients; to upon a specific request, to assess the ethical dimension and the feasibility of quality of care improvement programs developed at a local level; and to provide guidance upon request by institutional bodies on subjects of particular ethical or social relevance currently under debate.
Czarkowski et al. (2015). Hospital ethics committees in Poland. Poland. *Science and Engineering Ethics*	Clinical ethics committees	Quantitative, survey, selected hospitals, *n* = 176	To analyse the activity of HECs in Poland	There were different names for ethics services used, such as: advice committee for clinical ethics, ethical dilemmas committee, hospital’s ethical committee, hospital’s ethics committee, ethical team, ethical committee, ethical-medical team, and ethical team for geriatrics. Few Polish hospitals have HECs. Its structure, services and workload are not always adequate. In order to provide quality services by HECs, the development of relevant legislation, standard operating procedures and well trained members need to be implemented.
Førde & Pedersen (2011). Clinical ethics committees in Norway: What do they do, and does it make a difference? Norway. *Quarterly of Healthcare Ethics*	Clinical ethics committees	Questionnaires to all CECs in Norway (*n* = 39) regarding how the CECs were composed	The aims of this study are to learn how the national directives concerning the CECs have been followed by the local hospital trusts and to explore how the individual CECs in Norway function six years after the 2004 evaluation	The response rate was 79.5%. Committees were providing seminars for hospital employees. 26 of 31 of the committees’ activities consisted of the elaboration of ethical guidelines that discuss patient cases. Committees presented the patient’s perspectives through a patient representative in 91% of the cases. There is variation among the committees. This survey demonstrates that in spite of substantial challenges both ideologically and practically, the activity of the Norwegian clinical ethics committee system is substantial, and compared with the survey completed in 2004 the committees’ activities are increasing.
Larcher et al. (2010). Core competencies for clinical ethics committees. UK. *Clinical Medicine*	Clinical ethics committees	Theoretical paper	To engage the wider debate on whether CECs are the only, or indeed the most desirable model for the provision of ethics support and guidance in clinical practice	Provision of clinical ethics support may include consideration of individual cases, or debate on the ethical issues they raise; the education of health professionals on such issues; and ethical input into trust policy and guidance. It is accepted that these functions require the identification and analysis of ethical problems within a legal framework, if criticisms of lack of ‘due process’ are to be addressed. Since ethical support may be provided by individuals, small groups or committees, the core competencies identified are to be considered as “collective” in their application to a particular committee or group.
Pedersen et al. (2009). Barriers and challenges in clinical ethics consultations: The experiences of nine clinical ethics committees. Norway. *Bioethics*	Clinical ethics committees	Qualitative, semi-structured group interview of ethics committee members *n* = 24	To present the results from the qualitative section and provide an in-depth exploration of the barriers and challenges confronting the committees’ consultation services, as perceived by committee members	The committees functioned as a forum for the deliberation of ethically challenging questions arising in clinical work and provided decision-making support—primarily for the clinicians involved. The committees interviewed indicated that they sometimes had to find a balance between being perceived as supportive and non-judgemental by the healthcare personnel, and promoting certain standards and professionalism in moral deliberations, for example having open discussions of values that included all the involved parties, and having adequate documentation.
Schick & Guo (2001). Ethics committees identify success factors: A national survey. The Netherlands. *HEC Forum*	Clinical ethics committees	Mixed methods, national survey questionnaires *n* = 962, focus groups interview *n* = 2	To identify which factors are viewed as essential to success of a healthcare ethics committee by committee chairpersons and members	Both chairpersons and members ranked the categories of participation, communication, skills, confidentiality, client satisfaction, and composition of the committee members as most important. Chairpersons selected the multidisciplinary composition of the committee to be the most essential factor for the success of ethics committees, while members selected as most essential respect for others’ points-of-view.
Slowther et al. (2001). Clinical ethics support services in the UK: An investigation of the current provision of ethics support to health professionals in the UK. UK. *Journal of Medical Ethics*	Clinical ethics committees	Mixed methods; questionnaire surveys *n* = 2363, interview with chairmen of local research ethics committees *n* = 208	To identify and describe the current state of clinical ethics support services in the UK	Healthcare professionals, e.g., senior clinicians, managers, health authority members, and chief executives, believe some ethics support services are desirable. Clinical ethics support is at an early stage and needs to develop in the UK.
Slowther et al. (2004b). Development of clinical ethics committees. United Kingdom. *British Journal of Medicine*	Clinical ethics committees	Theoretical paper	To describe ethics committees within NHS and their purposes	The aim of committees is to facilitate ethical decision making by doctors and hospital policy makers. A national clinical ethics network has been formed to facilitate and coordinate high quality ethics support. The network aims to promote good clinical ethics support throughout the United Kingdom.
Slowther et al. (2011). Development of clinical ethics committees: A national survey. United Kingdom. *Clinical Ethics*	Clinical ethics committees	Quantitative questionnaire survey administered to the chairs of all 82 clinical ethics services registered with the UK Clinical Ethics Network	To describe the current provision of ethics support in the UK and its development since 2001	All services included a clinical ethics committee with one service also having a clinical ethicist. Lay members were present in 72% of responding committees. Individual case consultation has increased since 2001 with 29% spending more than 50% of their time on this. Access to and involvement in the process of case consultation is lower for patients and families than for clinical staff. There is wide variation in committee processes and levels of institutional support. Over half of the responding committees undertook some form of evaluation. Clinical ethics services in the UK are increasing as is their involvement in case consultation. However, there is significant variation in committee processes.
Wenger et al. (2002). Hospital ethics committees in Israel: Structure, function and heterogeneity in the setting of statutory ethics committees. Israel. *Journal of Medical Ethics*	Clinical ethics committees	Mixed methods, quantitative, cross-sectional national survey of general hospitals, *n* = 28, qualitative interviews, *n* = 8 with chairpersons of and physicians on ethics committees	To describe the current form and function of hospital ethics committees in Israel and the cases that they hear	Among the eight hospitals with 200 or more beds that have no ethics committee, two indicated that they have been unable to locate a qualified chairperson for an ethics committee. In two of the eight hospitals, individuals in hospital administration perform a form of ethics consultation. Many Israeli patients and clinicians do not have access to ethics committees.
Dauwerse et al. (2014). Implicit and explicit clinical ethics support in The Netherlands: A mixed methods overview study. The Netherlands. *HEC Forum*	Moral case deliberation	Mixed methods, survey questionnaires *n* = 2, focus groups *n* = 2, in-depth interviews *n* = 17	The purpose of this article is to investigate the prevalence of different kinds of CES in various Dutch health care domains, including hospital care, mental healthcare, elderly care and care for people with an intellectual disability	In The Netherlands, ethics committees are important vehicles explicitly for CES, especially in hospitals. A second important kind of CES is moral case deliberation, which can be found in half of Dutch health care institutions and in two-thirds of the institutions for mental health care. Ethics consultants play a minor role in all contexts of Dutch health care. Combining implicit and explicit CES is considered to be a good way to embed ethics integrally into the organization. This opens new perspectives on the meaning, positioning, and ownership of ethics in general and CES in particular.
Janssens et al. (2014). Evaluation and perceived results of moral case deliberation: A mixed methods study. Netherlands. *Nursing Ethics*	Moral case deliberation	Mixed methods, questionnaires *n* = 493, in-depth interviews *n* = 5, focus group meetings *n* = 3	To gain insight into what participants consider to be the value of MCD for themselves as professional care givers and for their organisation, with a specific focus on the contribution of MCD to care practice	The result showed that participants in moral case deliberation (MCD) evaluated MCD positively. In particular the atmosphere of the MCD sessions scored high, while organisational issues regarding MCD scored lower and merit further attention. Participants indicated that MCD has the potential to contribute to care practice by improving relationships among team members, generating more openness and fostering greater understanding for different perspectives. The relevance of MCD for care practice received wide acknowledgment from the respondents. It can contribute to the team’s cohesion as mutual understanding for one another’s views is fostered.
Gracia (2001). Moral deliberation: The role of methodologies in clinical ethics. Spain. *Medicine, Health Care and Philosophy*	Moral deliberation	Discussion article. Comparison of two methods from philosophical perspectives including utilitarian and principlism	To analyze two methodologies: the “dilemmatic” and the “problematic”	It is easier to reason than to deliberate. Deliberation is a difficult task and it requires many conditions, such as: lack of external constraints, good will, capacity to give reasons, respect for others when they disagree, an ability to listen, disposition to influence and to be influenced by arguments, and a desire to understand, cooperate and collaborate. This is the framework of a true deliberation process. Deliberation rests not on “decision” but on “commitment.” Within this framework, almost all existing bioethical methods can be useful to some extent.
Molewijk et al. (2008a). Teaching ethics in the clinic. The theory and practice of moral case deliberation. The Netherlands. *Journal of Medical Ethics*	Moral case deliberation	Evaluation survey *n* = 57 + *n* = 11, interviews *n* = 6, focus groups *n* = 2, participant observation	To present an alternative, contextual approach to teaching ethics, which is grounded in a pragmatic hermeneutical and dialogical ethics	Ethicists and healthcare professionals who are involved with moral case deliberation projects need to find balanced and reasoned answers to role questions. The theoretical background of pragmatic-hermeneutics and dialogical ethics provides a framework for dealing with those questions in a non-dogmatic way.
Molewijk et al. (2008b). Implementing moral case deliberation in a psychiatric hospital: Process and outcome. The Netherlands. *Medicine, Healthcare and Philosophy*	Moral case deliberation	Mixed methods, in-depth interviews with staff *n* = 3, questionnaire *n* = 69	(a) To describe the practice and the theoretical background of moral deliberation; (b) to describe the moral deliberation project; (c) to present the outcomes of the evaluation of the moral case deliberation sessions; and (d) to present the implementation process	The results showed that the moral case deliberations, the role of the ethics facilitator, and the train-the-facilitator program were regarded as useful and were evaluated as (very) positive. Healthcare professionals reported that they improved their moral competencies. They have developed skills to reflect on their work, and to create an atmosphere of dialogue instead of discussion and debate.
Molewijk et al. (2008b). Implementing moral case deliberation in Dutch healthcare-Improving moral competency for professionals and quality of care. The Netherlands. *Bioethica Forum*	Moral case deliberation	Mixed methods, in-depth interviews (*n* = 5) with healthcare professionals, questionnaire *n* = 220	To give a definition of MCD, to describe its theoretical background, to describe a 4-year MCD implementation project in a psychiatric hospital, to present the first results of a study on the quality and results of MCD sessions	The results of the 220 questionnaires of 50 MCDs showed that the MCDs were regarded as being very useful. The participants saw the relevance of MCD for their daily work and judged the quality of the dialogue positively. Their open, straight, constructive communication and moral sensitivity increased; their presuppositions, prejudices and automatic responses decreased. Future research needs to investigate what the long-term impact will be on the quality of care.
Molewijk et al. (2011a). Emotions and clinical ethics support. A moral inquiry into emotions in moral case deliberation. Netherlands. *HEC Forum*	Moral case deliberation	Qualitative, case discussion among participants with interdisciplinary profession, *n* = 20	To exchange practical experiences dealing with emotions within CES, and to develop practical suggestions for dealing with emotions in a suitable way	The case description shows that within clinical ethics support one needs to critically reflect on one’s emotions. By focusing on the emotion in the case, one learns how to deal with emotions in practice and integrate them in moral life. This study showed that emotions play a crucial role in moral life. Emotions should neither be followed instinctively, nor be discarded and put aside. A proper way of dealing with an emotion is finding the right middle ground between being overwhelmed and remaining untouched. Moral case deliberation can provide tools for dealing with emotions in clinical practice. This is not just a matter of rationally determining a balance. One has to be able to act in line with the right middle, and embody the appropriate attitude. Dealing with emotions is a matter of virtue and character.
Weidema et al. (2012). Enacting ethics: Bottom-up involvement in implementing moral case deliberation. The Netherlands. *Health Care Analysis*	Moral case deliberation	Qualitative, in-depth interviews *n* = 5, focus groups *n* = 1	To describe MCD implementation processes from the perspective of nurses who co-organize MCD meetings, so called “local coordinators”	Approaching implementation of ethics support activities like MCD from the perspective of local coordinators showed that organizing ethics support involves a lot of activities. These activities, like settling preconditions for a session, remain invisible when focussing on ideological considerations only. Local coordinators reveal important experiential knowledge on how to do ethics support such as MCD. For example, realising what the meaning of a word (like “moral case deliberation”) can do in practice. Local coordinators indicate, because of their practical involvement, apparent trivialities have impact on the progression of an MCD series. Ethicists initiating MCD should seriously take into account the organizational and practical side of the activity to be implemented. Initiatives are and should be translated into the particular context. Implementing ethics support activities, meaning and organizational culture are crucial.
Svantesson et al. (2014). Outcomes of Moral Case Deliberation—the development of an evaluation instrument for clinical ethics support (the Euro-MCD). Europe, *BMC Medical Ethics*	Moral case deliberation	Qualitative. Interviews with ethicist and ethics researcher (*n* = 13) and healthcare providers (*n* = 73) combined with explorative literature review	To develop a multi-contextual evaluation instrument measuring health care providers’ experiences and perceived importance of outcomes of Moral Case Deliberation	A European Moral Case Deliberation Outcomes Instrument (Euro-MCD) was developed. It consisted of two sections, one completed before a participant’s first MCD and the other after. The instrument contained a few open-ended questions and 26 specific items with a corresponding rating/response scale representing various MCD outcomes. The items were categorised into the following six domains: enhanced emotional support, enhanced collaboration, improved moral reflexivity, improved moral attitude, improvement on organizational level and concrete results.
Widdershoven et al. (2016). Ethical theory as part of clinical ethics support practice. The Netherlands, *The American Journal of Bioethics*	Clinical ethics support	Theoretical paper	To examine two issues: the role of ethical theory in the deliberation on ethical issues, and the relevance of ethical theory for facilitating deliberation	Ethical theories are relevant for perceiving and analysing moral problems in clinical practice, and for developing and justifying methods of CES. It can stimulate reflection and deliberation if it is directly related to practice addressing practical moral knowledge of the participants in the deliberation and fostering their moral work. Ethical theory is important in CES, not as an external source, but as an integral part of CES practice.
Bollig et al. (2015). Ethical challenges in nursing homes – staff’s opinions and experiences with systematic ethics meetings with participation of residents’ relatives. Norway and Austria. *Scandinavian Journal of Caring Science*	Ethics discussion	Mixed methods. Questionnaire *n* = 93	To investigate nursing home staff members’ opinions and experiences with ethical challenges. To find out what types of ethical challenges and dilemmas occur and are being discussed in nursing homes	The most frequent ethical challenges reported by the nursing home staff were: lack of resources, end-of-life issues and coercion. To improve systematic ethics work, most employees suggested ethics education (86%) and time for ethics discussion (82%). Of 33 documented ethics meetings from Austria over a 1-year period, 29 were prospective resident ethics meetings where decisions for a resident had to be made. Agreement about a solution was reached in all 29 cases, and this consensus was put into practice in all cases. Residents did not participate in the meetings, while relatives participated in a majority of case discussions. In many cases, the main topic was end-of-life care and life-prolonging treatment.
Forsgärde et al. (2000). Ethical discussion groups as an intervention to improve the climate in inter-professional work with the elderly and disabled. Sweden. *Journal of Interprofessional Care*	Ethical discussion groups	Quantitative, intervention study. ‘Experimental dwellings’ ethical group discussion *n* = 4	To improve the work climate in inter-professional groups	The small observed changes after intervention indicates that the intervention did not lead to the expected improvement in the work climate, but might also result from the chosen scales inability to measure complex social processes. The importance of inter-professional discussions about everyday skills and values is stressed.
Lillemoen & Pedersen (2015). Ethics reflection groups in community health services: An evaluation study. Norway. *BMC Medical Ethics*	Ethics reflection groups	Qualitative, focus group interviews *n* = 3	To evaluate systematic ethics reflection in community health groups	Ethics reflection groups focusing on ethical challenges from the participants’ daily work were found to be significant for improved practice, collegial support and cooperation, and personal and professional development among staff, facilitators and managers. Resources needed to succeed were managerial support, and anchoring ethics sessions in the routine of daily work. Ethics reflection is a valuable measure to strengthen clinical practice. Ethics reflection based on experiences and challenges in the workplace was described as a win–win situation.
Grönlund et al. (2016). Managing ethical difficulties in healthcare: Communicating in inter-professional clinical ethics support sessions. Sweden. *HEC Forum*	Ethics rounds	Qualitative, recorded audio and video of clinical ethics support *n* = 10	To describe the communication of value conflicts during a series of inter-professional CES sessions	In an open and permissive communication climate with guidance from competent leaders, professionals may stimulate each other to face their ethical difficulties, change their attitudes to situations, help each other to find alternative ways of handling situations, and further develop their professionalism.
Silén et al. (2014). Ethics rounds: An appreciated form of ethics support. Sweden. *Nursing Ethics*	Ethics round	Qualitative, interviews *n* = 11 with health care personnel	To gain a deeper understanding of how the ethics rounds were experienced and why the intervention in the form of ethics rounds did not succeed in improving the ethical climate for the staff	The staff experienced changes by participating in the ethics rounds in the form of being able to see things from different perspectives as well as by gaining insight into ethical issues. By listening to others during ethics rounds, a person can learn to see things from a new angle.
Sporrong et al. (2007). Developing ethical competence in health care organizations. Sweden. *Nursing Ethics*	Ethics rounds	Mixed methods, Qualitative, ethics round *n* = 3 and ethics theory lectures *n* = 3. Quantitative, questionnaire *n* = 259	To evaluate the impact on perceived moral distress after an education and training program in ethics, which included ethics rounds, for healthcare staff in different settings. To test the assumption that enhanced ethical competence would help to decrease reported moral distress, a prospective controlled study was set up	Ethical competence is a key factor in preventing or reducing moral distress. The results show that generally, there were differences in levels of moral distress between pharmacies and hospital departments. Ethics rounds may be seen as opportunities for ethical discourse, where participants jointly explore their own personal sets of values and seek to balance these with professional value sets. The ethics rounds method was also developed to strengthen the organizations’ ethical dimension.
Svantesson et al. (2008). Inter-professional ethics rounds concerning dialysis patients: Staff’s ethical reflections before and after rounds. Sweden. *Journal of Medical Ethics*	Ethics rounds	Quantitative, questionnaires *n* = 194	To evaluate whether ethics rounds stimulated ethical reflection	The ethics rounds did not seem to stimulate ethical reflection, but did extend perspectives regarding the patients and increased awareness of relations with other professions. The findings show the need for inter-professional reflective ethical practice, but a balance between ethical reflection and problem-solving is suggested if specific patients are discussed.
Svantesson et al. (2008). Learning a way through ethical problems: Swedish nurses’ and doctors’ experiences from one model of ethics rounds. Sweden*. Journal of Medical Ethics*	Ethics rounds	Qualitative, interviews *n* = 18	To evaluate one ethics rounds model by describing nurses’ and doctors’ experiences of the rounds	Positive and negative experiences were reported. Good rounds included stimulation to broaden thinking, a sense of connecting, strengthened confidence to act, insight into moral responsibility and emotional relief. Negative experiences were associated with a sense of unconcern and alienation, as well as frustration with the lack of solutions and a sense of resignation that change is not possible. In assisting healthcare professionals to learn a way through ethical problems in patient care, a balance should be found between ethical analyses, conflict resolution and problem solving.
Dörries et al. (2014). The impact of an ethics training programme on the success of clinical ethics services. Germany. *Clinical Ethics*	Clinical ethics service	Quantitative, online questionnaires *n* = 167	To evaluate long-term satisfaction with the Hannover Qualification Programme and its impact on clinical ethics services	The Hannover Qualification Programme (HQP) was evaluated as helpful and the responders were capable of applying their acquired skills. Most participants could contribute to the implementation of clinical ethics services. They were satisfied with HQP and with the degree of changes in their hospitals. Clinical ethics education had long-term effects on trainees and on their respective hospitals. Problems were mentioned more in the field of utilization than with implementation or quality of clinical ethics services.
Dauwerse et al. (2011). Need for ethics support in healthcare institutions: views of Dutch board members and ethics support staff. The Netherlands, *Journal of Medical Ethics*	Ethics support	Mixed methods, survey questionnaires *n* = 2, focus groups *n* = 2, interviews *n* = 17	To investigate the need for ethics support in Dutch health care institutions in order to understand why ethics support is not often used in practice and which factors are relevant in this context	There is need for ethics support. Reasons underlying claims that there is no such need include: aversion to innovations, negative associations with the notion of ethics support service, and organizational factors like resources and setting. The promotion of ethics support in health care can be fostered by focusing on formats that fit the needs of (practitioners in) health care institutions.
Lillemoen and Pedersen (2012). Ethical challenges and how to develop ethics support in primary health care. Norway. *Nursing Ethics*	Ethics support	Mixed methods, questionnaires, *n* = 323, focus group interviews, *n* = 2	To identify the frequency of ethical challenges and how distressed the various types of ethical challenges make the primary healthcare workers feel, how important healthcare workers in primary care think it is to better deal with these challenges and what kind of ethics support they want	The majority of primary healthcare workers in this study reported that they experience ethical challenges in their work. These challenges were closely related to professional and organizational circumstances, with the lack of resources, e.g., lack of staff and competence being the most prominent. The findings showed that the healthcare workers’ values clash with what they see themselves doing in their practice, such as hiding medication in food, tying patients to the chair or using force to clean the patient. These are the issues that are given less attention than, e.g., ethical challenges related to end of life.
Magelssen et al. (2016). Ethics support in community care makes a difference for practice. Norway. *Nursing Ethics*	Ethics support	Quantitative, online questionnaires *n* = 2. Responses in total *n* = 354	To study outcomes of ethics activities and examine which factors promote or inhibit significance and sustainability of activities	The participants of this study found the ethics project to be highly significant for their daily professional practice. Outcomes include better handling of ethical challenges, better employee cooperation, better service quality, and better relations with patients and next of kin. Factors associated with sustainability and/or significance of the activities were sufficient support from stakeholders, sufficient available time, and ethics facilitators having sufficient knowledge and skills in ethics and access to supervision. The facilitators who are responsible for the activities must receive sufficient follow-up and training in ethics deliberation methods and relevant topics in health care ethics.
Dauwerse et al. (2011). Goals of clinical ethics support: Perceptions of Dutch healthcare institutions. The Netherlands. *Health Care Analysis*	Clinical ethics support	Mixed methods, questionnaires *n* = 515. Focus group discussions *n* = 2. In-depth semi-structured interviews *n* = 11	To present results of systematic, empirical research on what key persons in Dutch health care institutions, consider the goals of clinical ethics support	The goals that were most often mentioned as important included: attention to ethical issues (98%), raising awareness of ethical aspects (97%), fostering ethical reflection (95%), improving quality of care (93%) and supporting employees (92%). Respectively 17% and 26% of the ethics support staff indicated that “to advise about ethical issues” and “to make ethical policy” should (absolutely) not be a goal of CES. The findings illustrate that respondents see good care as the overall goal of CES.
Porz et al. (2011). Theory and practice of clinical ethics support services: narrative and hermeneutical perspectives. Switzerland. *Bioethics*	Clinical ethics support	Theoretical paper. To introduce narrative and hermeneutical perspectives to clinical ethics support services (CESS)	To describe a threefold consideration of theory and show how it is interwoven with practice	A threefold account of the relationship between theory and practice based on narrative and hermeneutical approaches were discussed. The relationship between theory and practice took the form of a “hermeneutic circle.” Using theories to interpret experiences makes theoretical concepts clearer. It indicates our basic attitudes to our daily work by summarizing: (1) that we acknowledge our dependencies and responsibilities within the social sphere, and (2) that we believe that all human identities are constructed by means of narratives as (3) we perceive human beings as story-telling agents. In addition, (4) we emphasized our focus on fostering mutual understanding; (5) we acknowledge that understanding is mediated by language, words and concepts; and (6) we opt for taking personal and professional experiences seriously, making them accessible in dialogues, and learn from each other in changing perspectives.
Schildmann et al. (2013). Evaluation of clinical ethics support services and its normativity. Germany. European Clinical Ethics Network. *Journal of Medical Ethics*	Clinical ethics support	Theoretical paper. “Descriptive evaluation” and “evaluation of outcomes”	To provide an analysis of normative presupposition relevant to CESS evaluation	Evaluators should be explicit about the normative presumptions concerning the goals, purposes and perspectives regarding CESS and the respective evaluation criteria. The study concludes with a brief argument for more sensitivity towards the normativity of CESS and its evaluation research.
Schlairet et al. (2012). Clinical ethics support services: An evolving model. USA. *Nursing Outlook*	Holistic care continuum	Evaluation method of four-year family support team in a regional medical center	To describe a model for providing clinical ethics support services as a broad spectrum of care for management of conflict and ethically difficult situations in health care	For patients, their families, and clinicians over the course of this four-year evolution in meeting ethics-related needs, the Holistic Care Continuum with Clinical Ethics Support Services made available via Family Support Team members, yielded improvement.
MacRae et al. (2005). Clinical bioethics integration, sustainability, and accountability: The Hub and Spokes Strategy. Canada. *Journal of Medical Ethics*	Hub and Spokes Strategy	Qualitative; implementing the Hub and Spokes Strategy at hospitals *n* = 7	To explain the challenges of current clinical bioethics services and, in response to these, propose the Hub and Spokes Strategy	The Hub and Spokes Strategy overcomes the challenges related to specialization, workload, and peer support inherent in the lone clinical bioethicist model. The goal is to enhance awareness, knowledge and skills by building and supporting ethics capacity and networking throughout the hospital. It also strives to improve patient care and quality of staff work-life by integrating ethics into research, education, and clinical practice.

## Results

The results revealed four CES approaches that are available to support healthcare personnel who are dealing with ethical issues (Table 1). They comprised: clinical ethics consultation, clinical ethics committees, moral case deliberation, and ethics rounds/ethics discussion groups/ethics reflection groups, which we have combined together due to the similarity of their form and content. Although CES can be categorized into four main approaches, it is important to point out that due to a lack of firm definitions, it is difficult to draw distinct lines between them, which results in some overlap of the boundaries.

### Clinical Ethics Consultation

Clinical ethics consultation is defined as a set of services that generally occurs following requests from healthcare personnel, patients or their surrogates (Aulisio et al. 2000). It can also be performed routinely by a permanent body such as a hospital ethics committee (Reiter-Theil 2000; Tomazic et al. 2004). The consultation is provided by an individual or a small team of individuals in response to ethical issues (Adams 2009; Aulisio et al. 2000; Tarzian and ASBH Core Competencies Update Task Force 2013). Those who provide consultations have various professions, such as physicians, nurses, social workers or members of the clergy (McClimans et al. 2016). It is argued that the person(s) who provide the consultations are required to have certain skills and competencies in ethics, in order to support healthcare personnel in dealing with ethical problems (Aulisio et al. 2000).

Ethics consultations have been shown to help patients and personnel clarify ethical problems arising in daily health care practices and to improve collaborative decision-making (Fox et al. 2007; Tarzian and ASBH Core Competencies Update Task Force 2013). Ethics consultations may have the goal of improving quality of care for the patient and/or for solving certain aspects of ethical conflicts that occur between healthcare personnel, patients and next-of-kin (Aulisio et al. 2000; Paola and Walker 2006).

Beside requests concerning specific patient cases, ethical consultation services can provide educational activities in order to increase awareness concerning ethics in the clinic (Fukuyama et al. 2008) or to help deal with moral distress (McClimans et al. 2016).

In the US, there has been a movement to certify ethics consultants to assure that they possess key knowledge and skill competencies (Tarzian and ASBH Core Competencies Update Task Force 2013). Ethics consultants should possess a range of knowledge competencies that includes moral reasoning and ethical theory, relevant ethical codes, health law and local policies, and knowledge regarding the clinical context and staff and patient perspectives (Tomazic et al. 2004). In terms of skills, ethics consultants should have the ability and interpersonal skills to assess the nature of the ethical conflict by drawing on relevant ethics knowledge and “process” skills required to conduct clinical ethics consultation services effectively. In addition, a code of ethics has been developed by the American Society for Bioethics and Humanities, which identifies a set of professional responsibilities for those engaged in healthcare ethics consultation (Tarzian et al. 2015).

Ethics consultation services are multifaceted. There is no agreement regarding their core role worldwide and they vary in role and function depending on the country. For example, in Japan, ethics consultation services may prioritize the review of scientific and clinical research (Fukuyama et al. 2008) before case analysis and patient consultation (Adams 2009; Aulisio et al. 2009; Tarzian and ASBH Core Competencies Update Task Force 2013). Ethical consultation services can be used in specific ways, such as in response to requests for assistance in addressing uncertainty or conflict regarding a value-laden conflict of interest (Aulisio et al. 2000; Paola and Walker 2006). This can be between various stakeholders, such as patients, next-of-kin, healthcare personnel or the health organization (Adams 2009; Tarzian and ASBH Core Competencies Update Task Force 2013). The role of ethical consultation may be less specific, such as when consultations are triggered by the institution in order to educate health personnel in how to deal with moral distress, to improve ethical and moral qualities of decision-making and actions (McClimans et al. 2016), or to review research protocols (Fukuyama et al. 2008). Some ethics consultant(s), (depending on the country) even have the authority to make decisions or give advice/recommendations, whether alone or in agreement with next-of-kin or healthcare staff, as to the best course of action.

The idea that an ethicist/consultant with specific knowledge can assume the role of ethics expert and make judgments in ethically difficult situations has been supported by some (Aulisio et al. 2000; Tarzian and ASBH Core Competencies Update Task Force 2013). It has been criticized by others, who argue that while there is expertise in ethics, there is no such thing as an ethics expert (Adams 2009; Rasmussen 2011, 2016). Regardless of the contrasting positions described above, the approach of ethics consultation remains authoritarian, because while the consultation process is triggered by health personnel requesting a consultation, it is the consultants who have the authority and power (as a result of their position) to interpret the clinical ethics case (Agich 2001).

### Clinical Ethics Committees

A clinical ethics committee is typically a standing committee which functions as an independent institution or authority to provide a formal mechanism for dealing with ethical issues in clinical settings (Akabayashi et al. 2008; Aulisio and Arnold 2008). Generally, the members of clinical ethics committees have various professional backgrounds such as: bioethicists/ethics consultants, clergy, social workers, lawyers, nurses, physicians, psychologists, therapists and community representatives (Akabayashi et al. 2008; Schick and Guo 2001). The goals and responsibilities of the clinical ethics committee are to protect the rights, safety and well-being of the patient in the health care setting or human subjects in research projects (Borovecki et al. 2010; Gaudine et al. 2010; Slowther et al. 2011). In addition, they are to identify and analyze ethical issues in clinical practice (Larcher et al. 2010; Slowther et al. 2001), promote training and education of health personnel, and provide guidance upon request (Caminiti et al. 2011). They commonly respond to requests to address ethical issues related to ongoing as well as retrospective patient cases; identify ethical needs within clinical settings; support healthcare personnel, patients and next-of-kin (Førde and Pedersen 2011), find agreements and make decisions; and review research protocols (Fukuyama et al. 2008; Gaudine et al. 2010). Clinical ethics committees are involved in responding to ethical issues, such as informed consent to treatment (Borovecki et al. 2010; Caminiti et al. 2011). Sometimes they provide decision-making support (Pedersen et al. 2009) in end-of-life situations or the continuation of life support (Slowther et al. 2004b). Clinical ethics committees can also provide education, seminars, workshops and training in ethics for hospital employees (Borovecki et al. 2010; Caminiti et al. 2011; Pedersen et al. 2009). They can review research protocols (except in the United States, where separate committees deal with this process), provide ethical input into hospital policies (Pedersen et al. 2009) and create guidelines (Slowther et al. 2001). Additionally, they can give an “expert” opinion regarding issues, such as the provision of treatment against a patient’s will and the disclosure of medical information against a patient’s wishes when it might be deemed necessary (Wenger et al. 2002). Clinical ethics committees promote an ethical dimension to health care and generate possibilities for improvement in care quality (Caminiti et al. 2011; Czarkowski et al. 2015).

There is no formal legal or regulatory governing framework for clinical ethics committees, which is in contrast to research ethics committees worldwide (Larcher et al. 2010; Slowther et al. 2004b; Wenger et al. 2002). Clinical ethics committees vary in function, structure and goals worldwide, but there are some commonalities in terms of the provision of advice (Slowther et al. 2001) and recommendations concerning the best course of action or discussions that lead to a good decision-making process (Schick and Guo 2001; Slowther et al. 2004b). Some committees themselves assume the responsibility to develop and improve guidelines and policies regarding prospective ethical challenges in clinical practice, and others are mandated by the health care institutions to do so (Aulisio and Arnold 2008; Slowther et al. 2001). Clinical ethics committees seem to have formal authority and legitimacy (without generalizing) to provide advice and recommendations concerning ethical issues arising in healthcare institutions (Slowther et al. 2004b).

### Moral Case Deliberation

The approach of Moral Case Deliberation (MCD) has been described in several ways. It is said to consist of a collaborative, systematic reflection on real clinical cases (Molewijk et al. 2008a, b; Weidema et al. 2012); methodological reflection on concrete cases among healthcare professionals; and facilitator-led collective dialogue (Gracia 2001) of healthcare personnel who reflect on a concrete moral question connected to real cases in their practice (Janssens et al. 2014; Molewijk et al. 2008a, b; Weidema et al. 2012). The goal of MCD is to support healthcare personnel to manage ethically difficult situations in their clinical practice (Svantesson et al. 2014), and to enhance ethical reflection among healthcare personnel concerning ethical issues and thus improve the quality of patient care. In other words, MCD can help deal with concrete problems and help train healthcare personnel so that they improve their ethical competencies. MCD is a “deliberationist” approach to ethical issues that supports the idea that reflection over ethically difficult situations is vital, and aims to make health personnel aware of ethical issues as well as related theories and how they might be applied in practice (Widdershoven et al. 2016). It supports health personnel in managing ethical issues and making independent decisions from the standpoint that health personnel are entitled to make decisions about how to deal with issues in clinical practice (Molewijk et al. 2008a, b).

During an MCD session, which can last from 45 minutes to one day and is led by an external facilitator, participants reflect individually and collectively about the moral aspects of a particular patient case (Molewijk et al. 2011a). The collective group discussion is facilitated by an ethicist or someone trained in some kind of conversation method relevant to ethics (Molewijk et al. 2008a, b; Svantesson et al. 2014; Weidema et al. 2012). MCD sessions are led by a facilitator who has no authority to decide on or to provide/recommend suggestions concerning the best course of action (Molewijk et al. 2008a, b; Weidema et al. 2012). The facilitator’s main role is only to stimulate an ethical discussion (mutual reflection) and to illuminate the ethical aspects of the case (Dauwerse et al. 2014; Gracia 2001).

In an MCD session, health personnel take the initiative to discuss a patient's case that they have found ethically difficult to manage (Molewijk et al. 2008a, b; Molewijk et al. 2011a). Each person participates in the MCD on equal terms regardless of his or her job title and all voices are to be listened to and respected. During the MCD session different kinds of emotion, e.g., frustration, anger, sadness can be expressed (Molewijk et al. 2011a). MCD is similar in many ways to ethics rounds (described below), but it is distinctive because it uses theoretically based conversation methods (Janssens et al. 2014), such as the Dilemma method or Socratic Dialogue (Svantesson et al. 2014; Weidema et al. 2012). The Dilemma method has been used in the Netherlands with the goal of helping healthcare personnel seek consensus regarding ethical issues (Molewijk et al. 2008a, b, 2011a; Weidema et al. 2012). In contrast, Socratic Dialogue aims to help healthcare personnel develop ethical skills and a reflective attitude towards the ethical issues they experience in their everyday clinical practice (Molewijk et al. 2008a, b).

#### Ethics Rounds/Ethics Discussion Groups/Ethics Reflection Groups

These three approaches overlap with each other to some extent since they have commonalities in terms of how they are constructed as well as in their functions and goals.

Ethics rounds are a form of facilitator/ethicist-led reflection, which involves discussion of a particular patient’s medical and ethical issues (Grönlund et al. 2016; Silén et al. 2014; Svantesson et al. 2008). During ethics rounds, healthcare personnel from different disciplines reflect over a patient's case that they are finding ethically difficult to resolve (MacRae et al. 2005; Svantesson et al. 2008), or over ethical challenges related to professional and organizational circumstances (Grönlund et al. 2016; Lillemoen and Pedersen 2012). The goal of the ethics round is to stimulate ethical reflection and promote mutual understanding between professional groups (MacRae et al. 2005; Silén et al. 2014), particularly through listening to each other’s perspectives (Svantesson et al. 2008). Ethics rounds have been described as supporting healthcare personnel develop ethical competencies and gain insight into ethical issues (Silén et al. 2014; Sporrong et al. 2007). They help healthcare personnel to examine their own views and obtain a better awareness and understanding of their colleagues’ ways of thinking and acting (Grönlund et al. 2016; Sporrong et al. 2007).

Other kinds of ethics support alongside ethics rounds are ethics discussion groups and ethics reflection groups, which have been used by healthcare personnel in nursing homes to discuss issues regarding end-of-life care, lack of resources and coercion (Bollig et al. 2015; Lillemoen and Pedersen 2012). Ethics discussion groups have also been used to improve the work climate and job satisfaction among nursing staff (Forsgärde et al. 2000), while ethics reflection groups have been described as an approach in which healthcare personnel sit together and reflect over ethical challenges in their daily practice (Lillemoen and Pedersen 2015). Other related kinds of ethics support have been used to train and educate healthcare personnel to acquire skills needed to respond to ethical issues (Dörries et al. 2010; MacRae et al. 2005). Promotion of ethics support in health care can be fostered by focusing on the needs of the healthcare institution and using ethical theory to interpret the experiences of the healthcare personnel’s everyday work (Dauwerse et al. 2011; Porz et al. 2011). Ethics support facilitated by a person with knowledge in ethics has been shown to help staff handle ethical challenges, improve cooperation between employees (Magelssen et al. 2016), increase healthcare personnel’s awareness of ethical aspects, and improve relations between staff and patients/next-of-kin (Magelssen et al. 2016; Schlairet et al. 2012). To ensure that ethics support is improving the quality of care, evaluation is essential (Schildmann et al. 2013).

These approaches to ethics support are all characterized by the involvement of inter-professional healthcare teams in collective group discussions to encourage reflection over ethical issues that occur in clinical practice from the perspectives of the healthcare personnel themselves (Schlairet et al. 2012). A bioethicist or facilitator has the role of creating an equal atmosphere for everyone during the ethics rounds/discussion (MacRae et al. 2005). It is common that the bioethicist/facilitator receives some information concerning the case in advance, or asks the participants to prepare a case or an ethical issue to reflect over (Grönlund et al. 2016). The bioethicist/facilitator has no authority and does not act as an expert in ethics, nor do they provide advice about what to do, but only stimulates group discussion (Magelssen et al. 2016; Silén et al. 2014). One fundamental idea with these kinds of discussions is that the personnel may stimulate each other, enhance critical thinking (Svantesson et al. 2008), or change their attitudes regarding the situations. These discussions have the goal of helping personnel deal with moral distress (Sporrong et al. 2007), getting them to help each other and to cooperate in order to find alternative ways of handling situations, and to ultimately improve the quality of patient care (Dauwerse et al. 2011; Janssens et al. 2014). In these types of ethics support, healthcare personnel are considered to be qualified (Dörries et al. 2010) and legitimate decision-makers in regard to ethically difficult situations. Ultimately, making decisions or reaching a consensus regarding the best course of action in a particular case remains with the healthcare personnel (Silén et al. 2014; Sporrong et al. 2007).

## Discussion

The included articles (*n* = 54) covered a range of clinical ethics support. The study aim was to describe which clinical ethics support approaches are available to support healthcare personnel in clinical practice in terms of their construction, functions and goals. The existing literature clearly demonstrates the increased worldwide interest for clinical ethics support.

There are similarities and differences among the established approaches. In the first approach, clinical ethics consultation, the ethicist assists the healthcare personnel with patient cases where there could be issues regarding patient autonomy, informed consent, confidentiality, and surrogate decision making (Aulisio et al. 1998, 2000). Ethics consultation can sometimes be recognized as having an authoritarian “top-down” perspective (Adams 2013; Agich 1995; Aulisio et al. 1998), especially if the outcomes of the consultation are not beneficial for the patient or the healthcare personnel. Here the consultant has an influential advisory role and may propose solutions, or act as the primary ethical decision maker with respect to the outcome and the process (Aulisio et al. 2000; La Puma and Schiedermayer 1991).

The second approach utilizes clinical ethics committees; it has many similarities to clinical ethics consultation. In this approach, instead of an individual person with expertise in ethics, a group of “experts” assists healthcare personnel by providing advice or recommendations from an “expert” point-of-view (Dauwerse 2013; Hoffman 1991). Traditionally, both clinical ethics consultation and clinical ethics committees have focused on providing advice or recommendations to healthcare personnel in clinical practice (Cranford and Doudera 1984). In addition, clinical ethics committees have been involved in supporting patients and their next-of-kin. Sometimes patient representatives are included in ethics committees (Førde and Pedersen 2011) when discussing patient cases. However, there seems to be variation in function and role regarding ethics support in ethics consultation and ethics committees both between countries and even within them. There is still no clear universal consensus of what ethics consultation should or should not provide during a case consultation.

In a third approach to CES, MCD is sometimes described as a “bottom-up” approach, in which reflection starts from the healthcare personnel’s experiences of everyday ethical issues related to clinical practice (Molewijk et al. 2008a, b; Spijkerboer et al. 2016). This approach seeks to increase the healthcare personnel’s insight into their moral responsibility (Svantesson et al. 2008) and to broaden perspectives through reflection. MCD differs from clinical ethics consultation and clinical ethics committees in that it fosters dialogue on ethical questions and reflection on ethical dilemmas (Dauwerse 2013; Stolper et al. 2014) rather than on decision-making in ethically difficult situations. The reflection in MCD is usually guided by a facilitator (Molewijk et al. 2011a), who is trained in various conversation methods, e.g., the Dilemma method and Socratic Dialogue (Molewijk et al. 2008a, b; Plantinga et al. 2012). The facilitator in the MCD does not claim be an “expert” in ethics, but merely stimulates healthcare personnel by asking questions about the case like Socrates did in his time (Stolper et al. 2014).

Finally, there are ethics rounds/ethics discussion groups/ethics reflection groups that are closely related to each other in how they are facilitated and the content they discuss. For this reason, we have chosen to discuss all three together as one approach. This fourth approach is also closely related to MCD. In a previous study, MCD was used as an umbrella term for ethics rounds/ethics discussion groups and ethics reflection groups (Svantesson et al. 2014). Previous studies described the group reflections as being positively regarded by healthcare personnel (van der Dam et al. 2011, 2013; Verkerk et al. 2004). It was said to increase their awareness concerning ethically difficult situations they experience in their clinical practice (Lillemoen and Pedersen 2015). It also increased job satisfaction and was associated with lower burnout rates, while workplaces without reflection remained unchanged.

Reflection is the act of sharing experiences and narratives in which the person is receptive for personal development. It has also been described as a process of learning and representation (Moon 2013). A reflective conversation for healthcare personnel in practice is fundamental in order to provide quality services (Schon 2003). The primary purpose of reflection is not to solve an issue arising in everyday practice, but to increase awareness of the various aspects of the issue. A positive side-effect of reflection could be that it leads to the ability to solve an issue that occurs. Reflection supports the idea of an enhanced critical thinking process. According to Dewey (1933), the purpose of reflection is to process knowledge in order to get a deeper understanding of a phenomenon. In health care, the personnel reflect on ethically difficult situations in order to learn more of what it means to act or not act in a certain way. Therefore, clinical ethics support in the form of reflection is vital for personnel working in health care settings. Personnel benefit from approaches that can create an atmosphere where they can have the freedom to express their feelings and emotions related to a case they are struggling with (Molewijk et al. 2011b).

Clinical ethics support from a “bottom-up” perspective might give healthcare professionals opportunities to think and reflect on issues they are facing in their everyday work. A dominant “top-down” perspective could be a less risky approach if, and only if, it removes ethical responsibility from the healthcare personnel (Agich 1995; Hansson 2002). For example, if a consultant makes a decision, or gives advice or a recommendation that is not beneficial for either the patient or the personnel, but only beneficial from an economical perspective. If later on the consequences of that decision or advice/recommendation proved detrimental to the patient, the healthcare personnel involved could free themselves from guilt by placing the blame on the consultant. If a decision or recommendation was based on a “bottom-up” approach that involves the reflections of the healthcare personnel, they would need to assume greater ethical responsibility and perhaps wish to reflect more in such situations. Consequently, the status of a professional “expert” in ethics might lead to a risk, an undermining, or a challenge to the healthcare personnel’s personal autonomy, e.g., a limitation on their autonomy when dealing with ethical issues. According to Schon (2003), professional practitioners are specialists that encounter certain types of situations again and again in their daily work. They learn what to look for and how to respond to those particular types of situations (Schon 2003). Even though many ethically difficult situations are unique, repeating patterns can be found. Therefore, the ethical responsibility and choice of what to do should remain with the healthcare personnel in clinical practice (Hansson 2002). To permit someone from the outside to make a decision or give a recommendation in a particular situation could be risky (Hansson 2002).

### Strengths and Limitations

A strength of this study is that the conclusions are based on literature that allowed an analysis of the established clinical ethics support approaches worldwide. In this integrative review, both qualitative and quantitative studies as well as theoretical papers were included. An integrated review allows the inclusion of several methodologies and can take into account a broader range of studies to develop a more comprehensive understanding of a phenomenon (Whittemore and Knafl 2005). Another strength is that we have searched data systematically with Mesh terms as well as manually. There are limitations as well. It was difficult to decide which articles to exclude since there are no real definitions as to what clinical ethics consultation really is. We included articles that described a clinical ethics support approach empirically, or theoretical papers that discussed an established approach. It was difficult to decide where to draw the line between approaches aimed only at supporting healthcare personnel with ethically difficult situations and approaches that support healthcare personnel as well as patients and next-of-kin. For example, some clinical ethics committees do support the patients and their next-of-kin, while other clinical ethics committees do not involve patients and next-of-kin in their annual meetings or when considering different situations. Even though there are similarities in the “top-down” perspective of the clinical ethics consultation and clinical ethics committees, it does not mean they are similar worldwide. Clinical ethics consultation and clinical ethics committees in different countries can differ in their role and function as well. In addition, in this review we considered only English-language papers and it is possible that other methods of ethics support have been developed in other language cultures. Future studies need to focus on defining and characterizing what clinical ethics support actually is, how it should function and if it should function differently in different countries; and if so, what are the possible reasons since many of the ethical issues are similar globally. Lastly, an additional interesting avenue for future research would be to perform a large-scale study of what types of ethical support practitioners are receiving, if they are satisfied with it and their perceptions regarding any effects on patient outcomes.

### Conclusions and Implications

Clinical ethics support in the form of reflection is an important approach in order to deal with ethical challenges in health care settings. Traditionally, clinical ethics committees and ethics consultations have been the two approaches that are sometimes recognized (without generalizing) as having a focus on providing suggestions or recommendations to healthcare personnel from an expert point-of-view, and often with a basis in different medical principles or theories. Moral Case Deliberation (MCD) and ethics rounds/ethics discussion groups/ethics reflection groups seem to focus on fostering dialogue on ethical questions and ethical inquiries that are brought up by the healthcare personnel. It has been argued that approaches based on reflection may generate insight (Moon 2013). In MCD for example, reflection from a “bottom-up” perspective may support healthcare personnel to process their own thoughts and feelings. This might further help them become aware of other aspects they were previously unaware of in ethically difficult situations and how to deal with them in clinical practice. MCD is an explicit form of clinical ethics support that gives professionals support to improve their moral reflection skills (Dauwerse et al. 2014). Reflection in groups might in the long-term help healthcare personnel discover their own way of dealing with ethically difficult situations in order to act in the best interest of the patient.

To summarize, clinical ethics support from a “bottom-up” perspective might provide healthcare personnel with opportunities to think and reflect more than from a “top-down” perspective. While a “bottom-up” approach leaves healthcare personnel with the moral responsibility for their choice of action in clinical practice, a “top-down” approach risks removing that responsibility.
